# Book Reviews

**Published:** 1819-01

**Authors:** 


### CRITICAL ANALYSIS

### OF RECENT PUBLICATIONS,

IN THE

### DIFFERENT BRANCHES OF MEDICINE, SURGERY, &c.

*On the Nature and Treatment of Tetanus and Hydrophobia; with some Observations on a natural Classification of Diseases in general.* By ROBERT REID, M.D. Licentiate of the King's and Queen's College of Physicians in Dublin, Member of the Royal Medical Society of Edinburgh, &c. —Dublin, 1817. pp. 136.

THE obscurity which has reigned over the two diseases treated of in the present essay of Dr. Reid, gives an interest to every well-developed fact that seems to stand in any near relation to their more latent springs of action in the system. Until a late period, our knowledge of the nature of both these morbid states of the animal economy had been confined to a barren observation of their more obvious external characters. Our pathology of each had been gratuitous conjecture; our treatment,—not empirical, for that follows the track of ascertained utility,—but undirected fortuitous experiment.

A leading object with the author, in this publication, as appears from the Preface, is to "attract the attention of physicians to certain operations of the animal economy, which have not hitherto been taken into consideration in the treatment of diseases." The two affections which become the specific subjects of investigation in his work, appear to have been incidentally taken up as affording an elucidation of the principles which have to him seemed very satisfactorily applicable in a more general research into the morbid states of the human frame.

Looking at the nervous system as the medium of connexion betwixt the various organs of the animal machine, whereby they receive reciprocal excitement, and their several actions become associated, we must necessarily consider it to bear a most important part in all the functions of health; and not less important, doubtless, is the influence of nervous affection in every condition of disease.

Building on such a basis, Dr. Reid views the nervous system in the triple division into which it naturally forms itself in the human frame, as the ground-work of a distribution of the whole economy into so many distinct but associated systems. He says—

"When we inspect the anatomical structure of man, we observe the nerves distributed into three grand natural divisions. The first, supplying the organic viscera of the thorax and abdomen, and which, from its peculiar formation, is properly called the ganglionic system by anatomists. The second is enclosed in the bony canal of the vertebral column, and may hence be denominated the spinal system: this distributes its nerves to all the muscular parts of the body. The third, having its centre in the head, comes under the name of the cerebral system: to this belong the intellectual powers and the five senses.

"Comparative anatomy shows, that nature seems to adopt this division throughout the various individuals of animated being:—some are endowed with the first, and of course enjoy only the limited faculties peculiar to that system; others have the second superadded; but man, of all other animals, possesses the three in the highest perfection."

Following this distribution of the nervous system, the author proposes to arrange diseases under three general heads, which should be named according to the nervous system in which each is principally seated. Keeping in view the sympathies of the animal economy, by which one part extends its influence to others often most remote, and from which morbid excitements may co-exist in organs appertaining to separate divisions of the system,—we think there is sufficient evidence in the phenomena of diseases to admit that the distribution here proposed has a natural foundation in the economy of the animal frame. We see diseases presenting in their phenomena evident affection of one only of these individual systems into which it is proposed to distribute the actions of the whole frame. We have disease of the ganglionic viscera, wherein neither the cerebral nor muscular systems seem to participate; so have we derangements of both these latter, which show, for a considerable time, no influence on the functions of other parts of the system.

After explaining away an apparent analogy which might, at first view, be thought to exist betwixt the distribution here proposed and the common distribution of the functions into vital, animal, and natural, which is palpably widely at variance with a division founded on the distinct offices of certain portions of the whole nervous system, Dr. Reid proceeds to say—

"One of the most important circumstances in favour of a classification of diseases, according to the principles before stated, is, that by this view we obtain a clearer view of the balance of activity, which is necessary to constitute health. This view may be confined to definite limits, when we observe that, although the balance may be deranged in a particular organ only, or even a part of an organ of one system, yet, when weighing it with regard to the others, the whole of the diseased system must be taken into consideration. Thus, in acute disease of the liver attended with symptomatic fever, we cannot consider the functions of the liver alone; but must take the whole of the ganglionic viscera, as opposed to the cerebral. Experience shows that this has long been tacitly agreed to, as the treatment in these cases is by no means confined to the liver, but extends to the viscera belonging to the ganglionic system in general."

That undefined and inexplicable power in the animal economy, which is opposed to the extension of morbid actions, and which is implied in the designation of the "Vis medicatrix Naturæ," Dr. Reid thinks will be more within the scope of our intelligence, and even more amenable to control, under the view which he takes of the whole system. He gives his ideas on this point in the following passage:—

"When, however, we contemplate the operations of the different systems which constitute the human frame, we will (shall) perceive that each system is formed by nature to perform certain functions in the animal economy. But they are so intimately connected with each other, when conspiring to support the animal body as one whole and entire system, that the function of any one cannot be interrupted without having some influence upon the rest. The effect, then, of this influence must be, to excite or repress the actions of the different organs. We will (shall) find, however, in the investigation of diseases, that the systems which are not immediately engaged in the morbid state, will take on actions which are not only capable of resisting the effect of disease upon themselves, but often prove salutary, by restoring the healthy balance of activity throughout the whole. This tendency, then, is the long-celebrated Vis Medicatrix Naturæ, which we are enabled to point out with precision, and support or excite, during the treatment of diseases, by considering the operations of the animal frame according to the natural classification which is now proposed."

The difficulty which might be anticipated of arranging in their appropriate stations all the modifications of disease which offer themselves to our notice, is met by showing that, in establishing with as much precision as the actual state of our physiological enquiries will enable us, defined notions of the healthy functions of the nervous apparatus in each particular system, we shall be prepared to detect, with considerable accuracy, any marked deviation from this standard, and be enabled to refer such lesion of function to its proper portion of the subdivided system. The intimate relations and reciprocal dependencies of the several parts, with the graduated influences of each superior over the inferior systems, are essentially to be considered in estimating the affection of an individual organ.

The ganglionic system being that immediately subservient to the actions of organic life, though modified by, and dependent for many of its functions on the two other systems, from its importance and extension, claims the first notice in the investigation both of health and of disease. To its office belongs whatever regards the assimilation, the deposition, the removal, the excretion, of the material elements of the animal body. Disease involving the derangement of any of these functions must, therefore, be referred to the ganglionic system.

The spinal system embraces the muscular apparatus of the body; since the nerves which are distributed to these organs have their common centre of union in the nervous mass of the vertebral column. The individual muscles of the animal frame are local organs; the office of each is merely local; they are dependent on the common system for their organic action, but their individual functions are topical and isolated. It is by their nervous communications that they become parts of a common system. Through this pervading medium, their single actions are governed, and their correspondencies established. But the nervous structure furnished to the muscles has a central organ in the medulla spinalis, sufficiently distinct from the other portions of the nervous system to support the distribution and separate consideration which Dr. Reid proposes.

The spinal system of nerves has, however, other functions than that of guiding and combining the muscular actions and power: it is in reciprocal subserviency to the other systems of the common machine. The muscular nerves are essential to the local office of the nutrient system, since a muscle diminishes in volume after the interruption of its nervous connexion with the common centre. This separation, too, interrupts the calorific process in the part, dependent, perhaps, on the topical functions of nutrition and absorption; since the transmission of the circulating fluids is still maintained, but the temperature is not kept up in the portion deprived of nervous influence. Hence the author says—

"If we should observe that the action of the muscles was impaired, while the circulation of the blood continued free; or that the muscular substance became diminished, while the powers of assimilation remained perfect; we should have little hesitation in attributing the disease to the spinal system."

To the cerebral system, as the seat of the intellectual faculties, and the central source of the nerves of sensation, we must refer derangements of the functions of this class: but, from the intimate relation betwixt this and the ganglionic system, we shall perhaps find that many of its affections have their origin in sympathy with derangements of the latter; and that these sympathies are reciprocal.

Having premised this general view of a natural classification of diseases, which we have endeavoured to convey to our readers as explicitly as the brief exposition of the author has enabled us, and which we think stands commended to deliberate consideration by an intrinsic promise of practical usefulness, Dr. Reid proceeds to examine the diseases of Tetanus and Hydrophobia, on the principles with which this preparatory arrangement furnishes him.

Tetanus first engages his attention. Without dwelling on the history of the disease, which, as he observes, may be found in every author who has treated on the subject, he shows that, from whatever cause it may have arisen, there is little variety in the course of symptoms; and that the seat of morbid affection is almost exclusively limited to the muscular system. The viscera of the thorax and abdomen, he says, are little affected in their functions.

"Vomiting sometimes occurs, but generally does not continue. It is usual enough for the appetite of hunger to remain through the whole course of the disease, and what food happens to be taken seems to be regularly digested; the urine is regularly secreted, although sometimes retained, and is voided with difficulty and pain. When the spasms are violent, the pulse is contracted, hurried, and irregular; but the respiration is affected in like manner, and during the remission the pulse and respiration usually return to the natural state."

The intellectual functions and the senses are little disordered, till the long continuance of this violent disease has drawn the whole system into a fatal disturbance.

"By reflecting on these phenomena of the disease, we are led to observe that the thoracic and abdominal viscera are not primarily affected, and that the disease cannot take its rise in the nervous substance supplying these organs; for, were it so, these viscera must immediately take on diseased action. Hence it must be concluded, that the ganglionic system is not the seat of the disease. The same argument is applicable to the cerebral system, comprehending the intellectual powers and the five senses."

After observing that the cerebral and ganglionic systems are rather opposed to the disease than tending to participate in it, and that such parts of the muscular system as are more immediately subservient to the others are later in falling into the disease, the author says—

"Having thus explained how these two systems do not appear to be the seat of the disease, we must naturally conclude that it rests altogether in the other system, or that of the spinal canal. Every circumstance of the disease conspires to substantiate this idea. We observe that the only parts of the body which are engaged in the disease, from the commencement, are those constituted of muscles; but, upon dissection, there is not the slightest injury to be discovered in their structure. Now, we know that the nerves which are distributed to those parts, and are the proper stimulants to muscular action in the living body, all take their origin from the nervous system of the spine. It is natural to conclude that, as we cannot discover, in examination after death, any morbid change in the parts which are acted on by the disease, we should expect to meet with some change in the parts which afford the stimulus to muscular action: hence the disease must be seated in the nervous system of the spine. When we examine the bodies of those in whom the disease has proved fatal, we can no longer have any doubt that this is the fact."

Dr. Reid then refers to the opinions of several of the later writers on this disease, to show that none of them had viewed the disease in the same light with himself; nor had they, indeed, attained any but vaguely conjectural notions on the subject: but, as our readers will recollect, we have pointed out a conformity of opinions on this subject between several of the most eminent physiologists in different parts of Europe, who appear to have adopted them without being indebted to each other. Dr. Reid afterwards considers the morbid appearances which have occurred to him, and which have afforded the basis of his own pathology. Several cases have contributed to establish the opinion which Dr. Reid has formed on tetanus; but the one which seems to present the strongest evidence in support of his doctrine occurred in the person of a poor lad, aged 13, in whom tetanus came on after a severe burn in the toes, which had happened to him by sleeping near a lime-kiln, where he had sought to shelter himself from the inclemencies of a winter's night: the particulars of which were detailed in the last volume of our Journal.

In an Appendix is given another case, wherein fatal tetanic spasm came on in a soldier, exposed, whilst on duty, to a cold north-easterly wind blowing from the sea. Here also the chief, though not entirely exclusive, seat of disease was within the theca vertebralis.

"On carefully sawing the vertebræ, so as to lay bare the nervous mass of the spine, a great quantity of water was found between the vaginal ligament and the dura mater. A considerable quantity also appeared distending the latter membrane, as the fluid was contained between it and the pia mater. On raising the nervous mass, with its investing membranes, from the bodies of the vertebræ, there appeared some vestiges of inflammatory action about the upper dorsal vertebræ; but, on turning down the muscles and integuments which covered this part, it was found that a blister had been applied between the shoulders. Between the dura mater, however, and the bodies of the lumbar vertebræ, there was discovered a considerable effusion of watery blood into the cellular substance, which was exactly similar to what I have before represented to occur in cases of this nature. In this case there was also a considerable quantity of air contained in the vessels and cellular substance, along the whole length of the spinal canal."

Diseased appearances within the cavity of the spine have been observed in cases of tetanus, by various pathologists; yet until lately they failed to suggest to the observers the same view of the disease which Dr. Reid has here taken. It would be wrong to deduce, from a few apparently strong cases, any general conclusion as to the latent character of an obscure disease; but we must confess that the two instances which the author has given from his own experience, combined with the confirmative evidence of other observers, seem to afford an elucidation of the proximate cause of tetanus, so commensurate with the phenomena of the disease, that we can scarcely hesitate in subscribing to his opinions. Inflammation of the membranes of the medulla spinalis is the only adequate diseased action which can be traced in the cases submitted to consideration; and we can hardly scruple to recognise, in the effects of this action, all the affections manifested through the progress of the disease.

Exposure to great vicissitudes of temperature, Dr. Reid considers to be the chief predisponent cause to the disease: hence the liability to this affection of soldiers on active service, who, during their residence in quarters or in barracks, are secluded from that constant exposure to such alternations, which renders the constitution of the seaman comparatively exempt from the disease. Other circumstances, too, in the soldier's active duty, contribute more especially to induce disease of the spinal system: he carries on his back, through the fatigues of a day, his necessary baggage; and during the night, perhaps, is compelled to rest unsheltered on the damp ground. Here, surely, is ample preparation for the formidable disease to which we find him particularly exposed.

Dr. Reid observes, that the exciting causes of tetanus are commonly some injury of an external part, often very trivial; but that he has not been able to discover that the disease has arisen from internal injury, either of the alimentary canal or elsewhere.

In treating of the diagnosis of tetanus, Dr. Reid relies much on the difference to be observed in one of the earlier symptoms—trismus, which sometimes supervenes on gastric or intestinal irritation. When this last is the exciting cause of trismus, the author says, that the spasm is more continued, and the muscles which compress the lower jaw give to the finger examining them the sensation of greater uniformity of surface, than when this symptom forms one of the train which belongs to tetanus. In this latter case, the spastic muscles, alternately relaxed and contracted, have the feel of bundles of rigid cords.

Proceeding to the author's treatment of tetanus, we find him guided by the general views of disease which he lays down at the beginning of his work. He considers what is required to maintain the functions of the whole machine, in balancing and opposing the morbid and sound actions of the several nervous systems. In the healthy actions of the undisturbed systems, he finds a power opposed by nature to the extension of disease: this, therefore, he is jealous of diminishing. Under this view, he regards depletion by general bleeding as worse than inefficient; since by this, he says, we lower the power of the cerebral system, one of our most powerful antagonists to the morbid action, and thus lessen our means of resistance to the general destruction. Whatever weight we may attach to this consideration of the author's, it certainly claims deliberation, when the practice alluded to is in question, whether we shall be warranted in expending general strength (which may hereafter be essential in combating the disease, and which we have but limited means of repairing,) by a depletion that experience has not shown decidedly to be beneficially influential on the primary morbid action. Under the view, however, which we are disposed to take of this disease, we should decidedly anticipate advantage from, and be induced to adopt the practice, in an early stage of free topical detraction of blood by leeches along the whole track of the spine. This we should consider an useful preparative for the author's primary indication—blistering along the course of the vertebral column. When the disease seems disposed to put on a chronic form, the author says that there is every probability that advantage might be derived from a mercurial course, and the blister may be held in reserve, least the disease assume a more active character. Free cutaneous excretion, as a powerful detractor from muscular energy, he thinks should be excited, by a continued exhibition, every third or fourth hour, of from five to ten grains of the compound powder of ipecacuanha, assisted, when necessary, by James's powder.

The cerebral system, when threatened, must be supported by frequent and small doses of stimulants; of which the author commends Madeira wine as amongst the best. Without entering more fully (which our limits forbid) into

Dr. Reid's consideration of remedies which have been extolled, and which have proved too often inefficient in the treatment of tetanus, we have given concisely that course which he himself recommends; and with which, we must allow that we should be disposed very fully to coincide.

Of the other disease which Dr. Reid has undertaken to investigate, our notice must necessarily be brief. Indeed, as no novelty of fact, in elucidation either of the nature or treatment of hydrophobia is brought forward, we are only called upon to consider the peculiar views and reasonings of the author, as they tend or not to correct or enlarge our previous knowledge of an obscure and hitherto unmanageable disease.

After going through the history of the disease, and alluding to the obscurity of its pathology, the author says—

"However, if the symptoms of this terrible distemper are investigated, upon the principles which have been laid down, when speaking of the different nervous systems which constitute the human frame, the phenomena which have been a long time observed in the disease can clearly be marked out, and their true nature can be distinctly understood.

"It is evident, from the symptoms of the disease, that the ganglionic system is not engaged in morbid actions at the commencement. This system, therefore, is not primarily affected. When the disease has been induced by other causes than the bite of an enraged animal, it is never attended with delirium in the beginning, nor are the functions of the senses in any way disturbed. Hence it is evident, when terrific dreams have been observed, they cannot be considered as a symptom of hydrophobia, and must be attributed to the fears of the patient who has been bitten. The functions of the brain must, therefore, be exempt from the primary symptoms.

"The primary symptoms of hydrophobia are diminished muscular action, shown by the dullness and disinclination of the patient to move. Any exertion is attended with tremor, which gradually increases, until it amounts to irregular action. This is generally first conspicuous in the muscles of deglutition. The muscular parts are the first which exhibit any remarkable action in this disease. If, however, at this time the muscles be examined, there will not be found any marks of disease in their structure. It, therefore, evidently appears that the disease must be seated in the source from whence they derive the proper stimulus to their action. This has been shown, in a former part, to be the nervous system of the spine. Having once obtained this clue, the whole mystery of the disease can be easily developed."

Having thus determined, in his own opinion, the primary seat of morbid affection, the author's next enquiry is, whether defect or excess of energy be the characteristic of the affection; and, on comparison of the feeble, tremulous, and irregular excitement of the muscular system in this disease, with the rigid and tonic spasm of tetanus, he deduces the inference that the clonic spasms in hydrophobia are owing to the defect of nervous influence. "Hence," says he, "it is evident that the causes of this disease must act as sedative against the nervous system of the spine." Assuming then the point, that the functions of the spinal nervous system are importantly diminished in energy, the author proceeds to investigate the consequences which might, *à priori*, be anticipated in the general system from such morbid state. Emaciation, an inflammatory diathesis, modified by an oppressed respiration, and irregular and defective muscular action, are inferred from his pathology, and their existence demonstrated in the histories of the disease. The cerebral system is assailed by the re-action of a law of association, whereby remote parts, originally excited to peculiar actions through influence derived from the brain, if by any other cause they be drawn into that peculiar state, reflect on the cerebral system that impression which roused the original action. Thus tremor of the muscles, primarily brought on by fear, when induced by other causes, infects the mind with this depressing passion. But the cerebral system is more vitally injured by being robbed of its healthy stimulus in the blood; which, passing almost unchanged through the enfeebled and labouring organ of respiration, creeps through its torpid channels unstimulating, and rousing for its own propulsion, at each systole, a gradually sinking energy. The ganglionic system, unable to withstand the fatal influence, soon falls under the same depressing powers, and sinks in the common ruin.

The morbid appearances, on dissection, hardly offer decisive proof of the validity of this reasoning; nor are we prepared to say that they oppose it. Theories, attractive from their ingenuity and apparent harmony, prove too frequently delusive to be received with confidence on a bare suggestion. A single fact of subsequent discovery often destroys the most beautiful. It is the part, therefore, of wisdom to admit with caution what may attract only to betray.

It must suffice that we now slightly touch on the treatment which Dr. Reid's reasoning suggests to him as promising the best results. It must be directed, at the same time, to the three systems. To support the energy of the spinal system, the affections of which assume commonly an intermittent character, the author considers cinchona an appropriate remedy. He would also apply it to the part where the original wound had been excised. To keep up the vital property of the blood, deteriorated by the imperfect action of the pulmonary apparatus, oxygen gas should be inhaled several times in the course of the day. This, he conceives, will enable the blood to maintain the energy of the cerebral and ganglionic systems, and support their actions and power of resisting the disease, until the virus has had time to exhaust itself in the system which it first attacks. To invigorate the heart's action, by giving more stimulant power to the blood, whilst the vascular apparatus of the several organs was too much enfeebled to resist its impulse, would be to increase the chance of organic mischief. With the view, therefore, of lessening the injurious power of the circulating system, the author proposes to exhibit frequent doses of acetous acid; which has the property, he says, of accumulating in the venous system a larger proportion of the circulating fluid, thus leaving a diminished volume in the arterial tubes. Hence is lessened local pressure from action of the heart and arteries. It is said that numerous cases have lately occurred on the continent, which have been cured by this remedy alone.

The author concludes his volume in these words:—

"I have thus endeavoured to explain the nature and treatment of two diseases incidental to the spinal system. The one consisting in the extreme of increased action; the other being extreme diminished action. It is obvious that a variety of modifications may occur between these extremes of disease: to many of these nosologists have given distinct appellations. The treatment, however, in all must be founded upon the foregoing principles, according to the judgment of the practitioner in each individual case."

We have, on the present occasion, confined our observations on the work of Dr. Reid to almost a mere analysis of its contents; but, as we shall again consider this subject when we take up the work of Rachetti on Diseases of the Spinal Marrow, we defer till then the further remarks we may have to adduce.

*Minutes of Cases of Cancer, Part II.; being further Reports of Cancerous Cases successfully treated by the new Mode of Pressure: with some Observations on the Nature of the Disease, as well as on the Method of the Practice.* By SAMUEL YOUNG, Member of the Royal College of Surgeons; of the Medical and Chirurgical Society of London; and Surgeon to the Cancer Institution, &c. &c. 8vo. pp. 199. London, 1818.

BUT few medical works of modern production have been more generally read, or excited more attention from surgeons zealously devoted to the improvement of their art, than that of Mr. Young, in which he published the result of his enquiries into the nature of cancer. It is now thirteen years since the appearance of that treatise, and the author again comes before the public, to express his conviction of the correctness of his opinions, to enforce them by further arguments, and to display actual evidence of the efficacy of his mode of treating that formidable disease. The result of so many years' experience, from ample opportunities for observation, of a gentleman of acknowledged candour and considerable talents, has powerful claims on the attention of professors of surgery.

The opinions of Mr. Young have given rise to much discussion, and his mode of treatment has received a rather extensive trial under the direction of other surgeons; the results of which have neither terminated in an acknowledgment of the truth of his theory, nor of the efficacy of his practice. The principal objections to the former are—that cancer is a constitutional disease, or, considering it merely local, that it is accompanied with *specific* diseased action, which pressure cannot remove; and to the latter—its failure in the cases to which it has been applied. The arguments of Mr. Young, in opposition to the above-mentioned notions respecting this malady, are powerful and well-conceived; but as they, we presume, are well known to our readers, and have already been fully discussed, it only remains for us at present to consider "*the method of the practice by pressure.*"

Reflection, alone, would not lead us to form deductions at all satisfactory, even to our own minds, respecting the power of compression for the cure of cancer. Acknowledging the correctness of the opinions of Mr. Young on the nature of the disease, the success of the application of the remedy must appear doubtful in the extreme. Experience, alone, must here be our guide. That it has, under the superintendance of the author, cured many cases of disease, considered as cancer by surgeons of the first character for abilities and of the most extensive observation, is most satisfactorily evinced; but, when employed under the direction of other surgeons, it has not been equally efficacious.

The powerful evidence adduced by Mr. Young in favour of his mode of treatment induced the medical officers of the Middlesex Hospital to employ it in the cancer-ward of that institution. The following is the Report of the Medical Committee, appointed to take into consideration the results:

"That eight cases of open cancer (as it is commonly called), and eight cases of the schirrhous kind, have been submitted to the treatment by compression; some of them for several months, and others for a shorter period.

"That, in some of the cases of open cancer, combined with considerable œdema, the pressure was useful in lessening the volume of the tumor; but that it had not, even in a single instance, any salutary influence upon the specific nature of the disease. It frequently gave so much pain that the patients could not, after repeated trials, endure it, under any modification whatever; and often it appeared to hasten the fatal event.

"That, in schirrhous tumors, the disease advanced, rendering extirpation necessary in two instances: in six others, the disease passed into ulceration, assuming the usual malignant appearances, and terminating in death. Two cases still remain under the care of the respective medical officers.

"Your committee, however, although they cannot lay claim to the discovery of a specific, have still the consolation to believe that they have, in many cases, succeeded in obtaining great alleviation of suffering; such alleviation as might, perhaps, induce some speculative minds, less disciplined by experience, to conclude that they had at length succeeded in discovering a cure for cancer."*

The above evidence is certainly powerful; but it is by no means decisive, when we consider how impossible it is to employ this mode of practice in the wards of an extensive hospital, with the accuracy and care so insisted on by Mr. Young as absolutely necessary in order to obtain favourable results.

The ill effects which have been attributed to the use of the bandages are commented on by the author in the following terms.

"Nothing can be more ruinous to the object of this practice than the conversion, by careless or ignorant applications, of the proper pressure, into improper and partial stricture; or, on the other hand, where the diseased part, which ought to be the only one under the immediate influence of the pressure, is left untouched, with all the applications sliding over and rubbing its surface, and consequently producing irritation, while some other point of the body, generally the opposite side under the breast, across the stomach, or the side of the neck itself, is cut as if by a ligature. These *circumstances* are only *the abuse* of the practice; they ought never to happen, nor do they *ever* occur when the applications are made upon a right principle.

"Some of the most important branches of surgery depend upon the roller; and yet, in the first volume of Mr. John Bell's Surgery, there is an engraving of a gangrenous arm from bandaging. This

* *Surgical Observations*, by Mr. Charles Bell, Part i.

representation is given, not to shew the practical effects of bandages, but to exemplify, on the contrary, the consequences of *their abuse* under mismanagement, where, by careless or ignorant applications, the very object of their intention is not only perverted, but most dangerously reversed. So that, in the common use of the roller, in the ordinary duties of the surgeon,—duties by far the most useful, as justly pointed out by that author,—it is seen how easily benefits of the first practical importance, and which are supposed always to be at our command, are superseded by irremediable mischief, in consequence of ignorance or inattention; and precisely may the principle of that treatment, which forms the present object of attention, be so perverted."

An accurate and neat application of the bandage is, indeed, rarely witnessed in English surgeons; a fault which has called forth the censure of our rivals on the continent, who demonstrate to us the various and beneficial effects of this powerful remedy, which we are obliged to acknowledge, but which, from some cause or other, we can rarely obtain from the same means. In zeal for the improvement of our art, and attention to the comforts of the victims of disease, we certainly are not inferior to the French surgeons; but a desire to effect every thing by general means and bold and decisive measures, makes us too often inattentive to what we consider as trifling circumstances that do not influence general results, but which are in reality the points on which, in many cases, they absolutely depend. The foundation of inability for an accurate application, and want of knowledge of the use of the bandage, is often laid at the earliest period of the education of the English surgeon: he is taught by his instructors to leave it to patients themselves or their nurses, and thus to neglect the use of the most powerful of all instruments in the hands of an expert surgeon, but which requires more dexterity than all the apparatus of Scultetus. It is, therefore, not without a blush that we aver, we do not expect to see the hope of general success from the treatment of cancer by compression realized at the present period, although it were actually a remedy for that disease. We fear there are but too many bars to its proving very beneficial to the public, by the obstacles to its use becoming extensive, in the habits and circumstances of medical practitioners in general; the application of the bandage requiring that time and trouble which some may be indisposed to devote to it through disinclination, and others from a real want of leisure; and the instances, we believe, would not be rare where, with neither of these obstacles, the want of adroitness would prove prejudicial rather than useful.

The author has given very full instructions as to the manner of regulating the pressure in applying the bandages, which are too long to detail in this place; and, to give detached observations, would not convey to our readers a sufficiently correct idea of it, for practical use; we must, therefore, refer them to the work itself. The description occupies several pages, but does not subject the author to the charge of prolixity: if he have gone into any minute details, it arose from the laudable motive of being desirous to be clearly understood, that the advantages which he supposes his method to possess may be transmitted to others.

The general results of this mode of treatment are expressed by the author in the following terms:—

"The practice by the treatment of pressure, in cancerous complaints, and all states of tumor and diseased enlargement, admitting the application, is seen then to be regulated and intimately connected with the circumstances and nature of the disease, generally and individually. In some cases, a complete removal of all the disease, and restoration of the natural structure, is effected; in others, a thickened state of parts or indolent tumor will remain, after the removal of the more palpable and active disease; while, in other instances, the disease can only be partially diminished and kept at bay by constant resistance from the pressure, reducing the latter cases to the state of a ruptured patient, who constantly wears a truss. And even in this state, as remarked on before, a most evident and important advantage is obtained; since you diminish and keep that at a stand-still, the increase and advance of which forms the extremity of the disease, in which the sufferings, the comforts, and life, of the patient are involved.

"Such is the *rationale* of this practice, as confirmed by experience; and which will be generally admitted when the strangeness of novelty is worn off, when the subject is better considered, and the practice more adroitly understood and managed; and especially when a sense of that duty, which looks a little beyond the present, opens to the mind an uprightness of conduct, and fair dealing shall take place of that policy which, by detraction, perversions, and mis-statement, may obscure and injure, but never can ultimately *destroy*, the ends of truth."

The necessary length of the detail of the cases adduced by Mr. Young, to evince the beneficial effects of his mode of practice, prevents the possibility of our transcribing one or more of them for the information of our readers; but this we the less regret, as we feel convinced that the greater number of them will diligently peruse the work itself. Although they may not then be induced to form expectations of compression being a *cure* for cancer, they must acknowledge that it has stopped the ravages of the disease, even in its most destructive form, and rendered existence pleasurable which had previously been a mere susceptibility of agony.

*Medico-Chirurgical Transactions, published by the Medical and Chirurgical Society of London.* Vol. IX. Part I.—Longman and Co. 1818.

In the published records of a Society which boasts of names of merited celebrity in every department of professional science, we look with confidence for communications of value; and we are rarely disappointed. There is no institution, we believe, connected with our profession, in the United Kingdom, embracing within its own circle such ample means of collecting into its archives whatever may be deemed interesting or useful to medical research. From the abundant stores which are daily poured into this depositary of medical information, the council can have little difficulty in selecting, for the press, matter of general interest to the profession.

The first paper in the present volume is one of considerable value in a pathological view: the case marks strikingly the pertinacity of resistance in the vital power to the inroads of extended disease, when the organs immediately charged with the functions essential to life have not been involved in the primary morbid action. And yet we cannot but regret that the facts of this case were not rendered more serviceable to pathology, by pursuing the *post mortem* investigation to a satisfactory discovery of the original seat of disease.

I.—*A Case of Loss of Power over the Voluntary Muscles;* by John Bostock, M.D. Read Dec. 23, 1817.

Mr. H. betwixt 30 and 40 years of age, of good constitution, and of an active disposition, applied for advice in consequence of pain seated a little above the knee on the outside; not constant nor severe, but seemed to be increasing, and had induced a degree of stiffness in the limb that interfered with any unusual exertion. Some months before, he had slipped backwards down the stairs of a ship's cabin. A slight bruise seemed to be the only consequence, and he himself attached no importance to the circumstance. After two months, his power over the limb was perceptibly diminished; not being able to raise it sufficiently, and dragging it in a certain degree in walking. The pain advanced rather higher, but still not constant nor acute. During the next two months, the complaints slowly increased; pain not much more severe: sensibility of the limb to external impressions not impaired, nor its natural temperature diminished; but the command over its motions gradually lessened. During this time, no pain in the trunk; nor, upon very minute examination, could any disease be detected in the spine,

General health became a little impaired; appetite declined; bowels irregular, and a tendency to hæmorrhoidal affection.

After another interval of about two months, the other limb was perceived to be affected: he lost, in some degree, the command over it, and felt shooting pains about the knee and hip-joint. A new symptom, peculiarly distressing, now appeared: when he had been sitting or lying for some time, and first put his feet to the ground, a sensation darted through the legs, something similar to what we experience when the limbs are said to be asleep. This caused him to draw them up with an involuntary jerk, and, for a few moments, he felt very acute pain from this kind of exertion. There was, however, no numbness of the limbs at other times, and, when they were once firmly placed on the ground, the darting pain was no longer felt. Shortly after this, a difficulty in the articulation of particular words took place; and for the next four months the affection of the speech and limbs continued to increase, so that he became unable to move without assistance, and his power of utterance was nearly lost: bowels very irregular, and some degree of emaciation. About eight months from the commencement, pain in the back of the head and neck first occurred, and was described as an acute, deep-seated, darting sensation. It recurred in paroxysms at uncertain intervals, and was in some degree relieved by lying down. After one of these attacks, all the other complaints were aggravated, and the functions considerably disturbed. The general affections advanced with a slow but regular progress; and Mr. H. now began to experience a stiffness in his hands and arms, which at first prevented him from writing, and at length from performing the common offices of life. Some time after the commencement of the paroxysms above described, deglutition became sensibly affected, and soon after there was loss of power over the muscles of the jaws and the neck. During the next six months, his complaints advanced to a state of complete helplessness. Motion of the lower extremities and power of articulation entirely lost; the action of the hands and arms very nearly so; deglutition extremely difficult, and the jaws could only be opened to admit a small tea-spoon. His head fell down upon the chest, unless supported; saliva flowed constantly from the mouth, and he was frequently almost suffocated with mucus, or convulsed with ineffectual efforts to expel it. Occasional violent shooting pains in all the limbs; and the whole body so rigid, that not only was he unable to perform any motion without assistance, but it required considerable force to bend his body, so as to place him in a chair when he had been in bed, or to extend him in bed after he had been for some time sitting in his chair. His nights were restless, and one posture soon created great uneasiness. A large part of the day was occupied in taking in, by very small quantities and slowly, sufficient nutriment to satisfy hunger. There was no numbness nor insensibility of any part of the body, either to mechanical impressions or to changes of temperature; and all the external senses and the mental faculties remained uninjured: he exhibited considerable ingenuity in contriving methods of communicating his ideas, and at times manifested even a degree of cheerfulness.

For six months longer, the rigidity of the limbs increased, the pains became more violent, weight more harrassing, the difficulty of deglutition every day threatening its total suspension, nights were more restless; and yet the senses, both mental and corporeal, remained unimpaired. For some time before his death, the upper extremities, as well as the lower, were entirely motionless.

About three months before his death, in addition to the ordinary and now constant pains, Mr. H. had occasional paroxysms of a more violent kind. As far as his description could be understood, they commenced with an acute throbbing, which darted down the back of the head and neck, terminating in a complete rigidity of the whole body, and a temporary suspension of the faculties. He was always much debilitated by these attacks, and obviously worse after them: it was immediately subsequent to a violent seizure of this kind that he expired. A few days only before his death, his sight became indistinct, with some degree of strabismus; but there was no other affection of any of the functions connected with the nervous system, and the intellectual faculties remained unimpaired to the last. The state of the spine was examined several times during the course of the disease, (Dr. M'Cartney and Mr. Christian, of Liverpool, assisting,) but nothing morbid could be discovered in its form or condition.

After death, Dr. Bostock says, that the examination was begun by a most minute scrutiny of every part of the brain, without meeting with any appearance which could be considered as morbid. The basis of the cranium, and the cervical vertebræ, were removed for a more minute dissection at leisure; when, after a very careful investigation of the whole, every thing was found apparently in a natural state. The author goes on to say—

"After a very accurate survey of every part, we thought that we observed a slight furrow across the spinal cord, as if it had been compressed by a transverse ligature, and this in the place where it passes under the ring of the atlas; and, upon attentively noticing this part of the bone, it appeared a little thickened, and of a yellowish colour. These appearances, however, were not very distinct, and the change of structure existed in so slight a degree, that it would probably have escaped observation had any other morbid derangement presented itself to our notice."

In his review of the case, Dr. Bostock observes, that perhaps the most remarkable circumstance is the want of correspondence between the degree of disease and the morbid state of the parts, as discoverable upon dissection. This certainly, as the facts stand before us, is most remarkable; but we can scarcely bring ourselves to believe that we are fully prepared to speculate on this flagrant disparity of cause and consequence. We cannot persuade ourselves that the scrutiny has reached the real seat of morbid action. Here is a morbid affection progressively invading the whole of the muscular system. It does not attack an individual or a contiguous number of these organs, but involves systematically the whole motive apparatus in one scheme of destruction. The cerebral functions are untouched; the viscera of the thorax and abdomen are not primarily interested in disease. Hence we should infer that the mischief has its source in the common bond of muscular function—the nervous system of the spine. Preceding any manifestations of disease, an injury had been sustained, very sufficient for the excitement of morbid action within the canal of the vertebral column, of a character, it might be, not to attract immediate attention to its seat; for we know how insidious is the progress of slow disease. Pain and muscular affection first declared themselves in one of the extremities supplied with nerves from the lower portion of the spinal mass. Presently the fellow limb becomes similarly affected. Disease slowly propagates itself through the muscular parts, whose nervous communication is with the higher portions of the vertebral column. All the phenomena of the disease, as they present themselves to us, seem to be dependent on injury to parts contained within the tube of the vertebræ; and we conceive that disease, to this extent even, might be going on there, without its being cognizable by external examination. If the structure of the vertebræ or the ligaments were uninjured, and there were no disorganization of external parts, what should we have within our reach that should indicate, to external touch, a morbid state? But, without dilating further on a history which cannot but be considered as of great interest, we will only repeat our expression of regret that the restraint which in most cases unfortunately limits the investigation of morbid states should, in this instance, have operated to the prevention of a discovery of the real cause of such extended organic derangement, and so much individual suffering.

